# Co-Crystalline Solid Solution Affords a High-Soluble and Fast-Absorbing Form of Praziquantel

**DOI:** 10.1021/acs.molpharmaceut.2c00984

**Published:** 2023-03-08

**Authors:** Chiara Cappuccino, Enrico Spoletti, Fiammetta Renni, Elisabetta Muntoni, Jennifer Keiser, Dario Voinovich, Beatrice Perissutti, Matteo Lusi

**Affiliations:** † Department of Chemical Science and Bernal Institute, University of Limerick, Limerick V94 T9PX, Ireland; ‡ Department of Chemical and Pharmaceutical Sciences, University of Trieste, 34127 Trieste, Italy; § Department of Drug Science and Technology, University of Turin, 10129 Turin, Italy; ∥ Department of Medical Parasitology, Swiss Tropical and Public Health Institute, 4123 Allschwil, Switzerland; ⊥ University of Basel, Basel 4003 Switzerland

**Keywords:** praziquantel, solid solutions, mixed crystals, tartaric acid, malic acid, mini-capsules, chirality

## Abstract

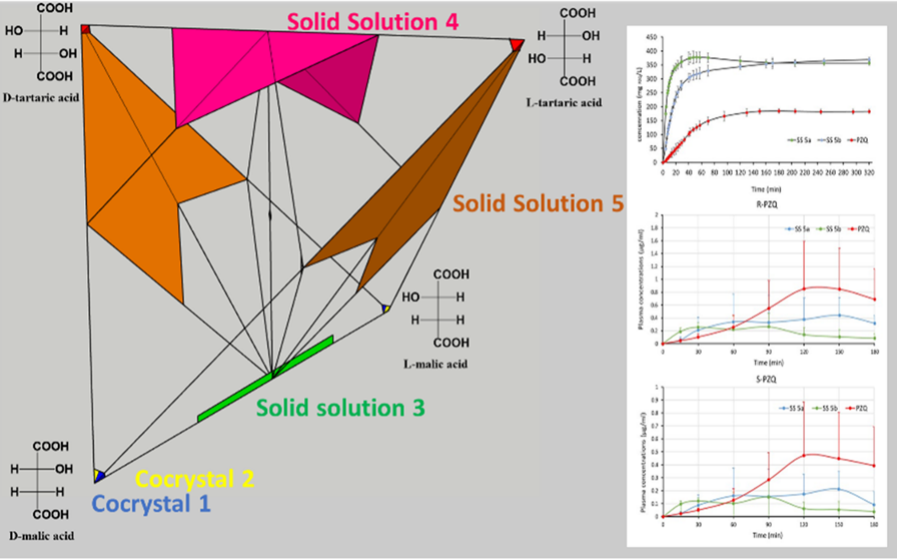

Praziquantel (PZQ) is a chiral class-II drug, and it is used as a racemate for the treatment of schistosomiasis. The knowledge of several cocrystals with dicarboxylic acids has prompted the realization of solid solutions of PZQ with both enantiomers of malic acid and tartaric acid. Here, the solid form landscape of such a six-component system has been investigated. In the process, two new cocrystals were structural-characterized and three non-stoichiometric, mixed crystal forms identified and isolated. Thermal and solubility analysis indicates a fourfold solubility advantage for the newly prepared solid solutions over the pure drug. In addition, a pharmacokinetic study was conducted in rats, which involved innovative mini-capsules for the oral administration of the solid samples. The available data indicate that the faster dissolution rate of the solid solutions translates in faster absorption of the drug and helps maintain a constant steady-state concentration.

## 1 Introduction

Praziquantel (PZQ) is the drug of choice for the treatment of schistosomiasis, a neglected tropical disease that affects 250 million people worldwide. Due to its low solubility and high permeability, PZQ is classified as a class II drug in the Biopharmaceutics Classification System. The drug is administered as a racemic mixture of two enantiomers, even though pharmaceutical activity relies mainly on the levo isomer [R-PZQ or (−)-PZQ], and a high dose (40 mg/kg) is required to ensure the desired plasma concentration.^1^ For instance, pediatric patients, the most affected by schistosomiasis, are administered several 600 mg tablets for a single treatment.^1^ The large volume of racemic tablets makes them difficult to swallow, which decreases patience compliance and causes many cases to be untreated.^2^ Smaller dose forms of pure R-PZQ are currently under development but considerably more expensive.^1^ Alternatively, a dose reduction might be achieved through the use of a more soluble form with increased absorption.

Common strategies to increase drug solubility employ cocrystallization and amorphization,^3,4^ where the cocrystal is referred to any multicomponent molecular crystal with a fixed (Daltonian) stoichiometry.^5^ Recently, racemic separation of PZQ via cocrystallization^6,7^ and its inclusion into mesoporous silica^8^ have also been investigated. These studies give some insights into the diversity of crystal packing available for the drug, which presents polymorphism and disordered structures.^9−12^ Many hydrates,^13^ solvates,^14^ and cocrystals of PZQ are also reported, especially with diverging dicarboxylic acids.^15^

It is established that the dissolution profile of a crystalline material is a function of its lattice energy.^16^ The latter depends on the overall molecular interactions, structural defects, and impurities, which can be controlled through the realization of a solid solution.^17,18^ An increasing number of examples show that solid solutions can be obtained in spite of long-established Rume-Rothery^19^ and Kitaigorodsky^20,21^ rules when the host structure is sufficiently flexible (i.e., shallow lattice energy) to accommodate the desired guest molecule. The cases of cortisone/hydrocortisone^22^ and tartaric acid (TA)/malic acid (MA)^23^ demonstrate that even molecules with different H-bond capability can form solid solutions.

Here, solid solutions of PZQ are investigated by crystallizing the racemic drug with MA and TA in enantiomerically pure, scalemic, and racemic stoichiometry. Therefore, instead of searching for yet another co-former, as usually happens in cocrystal screening protocols, we pursue property optimization in a single mixed (co)crystal.^24^ Ideally, the mixed crystals would enable a variation of the number (and orientation) of hydrogen bonds that are responsible for the overall lattice energy. As a consequence, thermal stability, solubility, and bioavailability of the solid product may be optimized.

## 2 Materials and Methods

European Pharmacopoeia (EP) grade PZQ [(11b*RS*)-2-(cyclohexylcarbonyl)-1,2,3,6,7,11b-hexahydro-4-hpyrazino[2,1-*a*]isoquinolin-4-one] was a kind gift from Fatro S.p.A. (Bologna, Italy). l-MA, l-TA, and water HPLC were commercially available from Sigma-Aldrich Company. d-MA and d-TA were commercially available from Acros Organics. Ethanol 96% was ordered from Honeywell Company.

### 2.1 Mechanochemical Syntheses

Polycrystalline multicomponent phases were prepared by liquid-assisted grinding of 0.2–0.4 mmol of racemic PZQ with MA and TA in the desired stoichiometry. For example, 0.2 mmol (62.5 mg) of PZQ, 0.1 mmol (13.4 mg) of l-MA, and 0.1 mmol (15.0 mg) of l-TA for PZQ/S-PZQ/l-MA/l-TA (1:1:1:1). A summary of the synthesis is provided in Table S1. All the mixtures were ground in the presence of two drops (100 μL) of ethanol for 120 min in a Retsch MM400 mixer mill (Retsch GmbH), operated at a frequency of 20 Hz; a 5 mL agate jar was used, with one agate ball 10 mm in diameter.

### 2.2 Powder X-ray Diffraction

All diffraction patterns were recorded on a PANalytical EMPYREAN diffractometer system using Bragg–Brentano geometry and an incident beam of Cu Kα radiation (λ = 1.5418 Å) in the 2θ range between 3 and 40° (step size: 0.013°; time/step: 30 s; Soller slit: 0.04 rad; divergence slit: 1/9; anti-scatter slit: 1/4; 45 mA × 40 kV). Room temperature scans were performed on a spinning silicon sample holder.

The diffraction patterns used for the structural resolution were collected in the 2θ range between 5 and 60°, with a time/step of 120 s and a Soller slit of 0.02 rad; three consecutive repetitions of the same measurement were collected and merged to obtain an optimal signal/background ratio.

### 2.3 Structure Solution and Rietveld Refinement

Powder diffraction data were analyzed using software EXPO2014.^25^ The unit cell parameters were found using the N-TREOR algorithm, from the 25 peaks chosen in the 5–35° 2θ range of the diffraction pattern (CCDC deposition number 2205816–2205817). Chebyshev and Pearson VII functions were used to fit the background and the peak shape, respectively. The crystal structures were solved by a simulated annealing and subsequently improved via a Rietveld refinement. To avoid incorrect molecular conformations during the solution and Rietveld processes, some geometrical restrains were placed on the free torsional angles and H-bond distances between the two coformers; an anti-bumping restrain has been added to avoid the atom collision. The molecular models used for the simulated annealing were preemptively optimized using MOPAC16.^26^ Crystallographic and refinement details are provided in the Supporting Information.

### 2.4 Thermal Analysis

Differential scanning calorimetry (DSC) measurements were performed on TA DSC Q-2000 under nitrogen stream (50 mL/min). Four–6 mg of ground powder accurately weighed was hermetically sealed into aluminum pans and heated from 20 to 200 °C, at a 20 °C/min heating rate.

### 2.5 Saturation Solubility

The solubility of the samples was analyzed by preparing saturated solutions of each sample at pH 7.4 in phosphate buffer, according to EP: 250.0 mL of 0.2 M potassium dihydrogen phosphate to 393.4 mL of 0.1 M sodium hydroxide. Such pH emulates the enteric conditions under which PZQ dissolves and is absorbed. The solutions were kept under agitation in the dark at 25 °C for 24 h, as this lapse of time had been previously determined to be adequate for equilibration. After filtration (pore size 0.45 μm), the PZQ concentration was quantified at a wavelength of 263 nm by UV analysis, performed with a UV-1800 Shimadzu UV–vis spectrophotometer.

### 2.6 Dissolution Kinetic Tests

Dissolution kinetic tests (DKTs) were performed in 75 mL of water at 37 °C. Notably, the solubility of PZQ is known to be independent from pH variation which might be caused by the presence of MA and TA.^27^ To use the in vitro dissolution as a prediction of the in vivo performance of each sample, sink conditions were not maintained during dissolution in order to build up supersaturation, which commonly occurs under finite-volume conditions in the gastro-intestinal tract, and to allow possible events such as nucleation, crystallization, and precipitation to proceed. Hence, at time zero, a suitable amount of the sample to give 60 mg of PZQ was added to the dissolution medium. Each DKT lasted for 60 min. During analysis, uniformity conditions were guaranteed by using an impeller (rotational speed 200 rpm).^28^ The determination of the PZQ concentration was performed by using a fiber-optic apparatus (HELLMA, Milano, Italy), which was connected to a spectrophotometer (ZEISS, Germany, wavelength 263 nm, the maximum PZQ absorption). Prior to quantification, a stock solution was prepared with 1,6 mg/mL PZQ in distilled water containing 5% methanol. Five dilutions were made starting from this mother solution, and experimental absorbance values were measured by the UV spectrophotometer (ZEISS, Germany) at 263 nm (*R*^2^ = 0.991). This technique allows the in situ determination of the concentration of a substance without perturbing the dissolution environment and often overcomes the problem connected to drug concentration measurements in the presence of generated solid particles. MA and TA maximum UV absorption occurs at a lower wavelength not interfering with PZQ quantification. A Tyndall–Rayleigh scattering correction was applied to the recorded spectra to exclude the scattering, occurring at every wavelength, of undissolved drug particles or excipients and to obtain the absorbance of the dissolved drug only. Each sample was tested in triplicate to obtain the mean concentration value at each time point. S.D.: did not exceed 5%.

### 2.7 In Vivo Pilot Tests on Animal Models

The procedures conformed to the institutional guidelines on animal welfare of the Ethics Committee of the University of Trieste (Directive 2010/63 UE) and international guidelines, with all effort being made to minimize the number of animals and their discomfort (3R guidelines). Furthermore, procedures adhered to ethical standards for humane treatment of experimental animals established by the ethical committee of the University of Trieste and authorized by the Italian Ministry of Health (1120/12).

Three different samples, as a powder, were administered by gavage: **SS 5a**, **SS 5b**, and commercially available PZQ, with a dose of 89.5 mg/kg.

For the oral administration, hard gelatin capsules size 9 (Torpac Europe BV, Heerlen, The Netherlands) were filled with the appropriate amount of powders, as they provide a suitable method for the oral dosage to laboratory rats weighting 310–330 g by using a Torpac dosing syringe. This procedure eliminates validation of suspension/solution homogeneity and vehicle excipient absorption effects. Before starting the study, the animals (Wistar male rats, Charles River Italia, Milan, Italy) underwent a short training period of 3–4 days: the insertion of the syringe tube did not appear to cause undue discomfort to the rats or tissue damage. The procedure, which can be performed rapidly by trained personnel, is ideally suited for dispensing solid materials to fully conscious animals.^29^

The rats, with free access to water, were fasted 12 h before the experiment. At scheduled times (15, 30, 60, 90, 120, 150, and 180 min) after administration, blood samples were collected into heparinized tubes by means of a catheter surgically positioned in the jugular vein.^30^ Each experiment was performed on four rats, for **SS 5b**, **SS 5a**, and commercially available PZQ. Blood samples were centrifuged at 17,000*g* for 5 min, and then, 0.25 mL of plasma was added to 0.075 mL of 10% trichloroacetic acid solution for precipitating plasma proteins and centrifuged at 55,000 for 5 min. Finally, 0.025 mL of pH 5 buffer (5 M acetate buffer) was added to the supernatant.

### 2.8 LC–MS/MS Plasma Analysis

The liquid chromatography tandem mass spectrometry (LC–MS/MS) method used for the quantification of PZQ enantiomers in this study was adapted from an already validated method for human plasma using a lower sample volume and a slightly changed matrix,^31^ already described in previous publications.^32^

## 3 Results and Discussion

**Figure 1:**
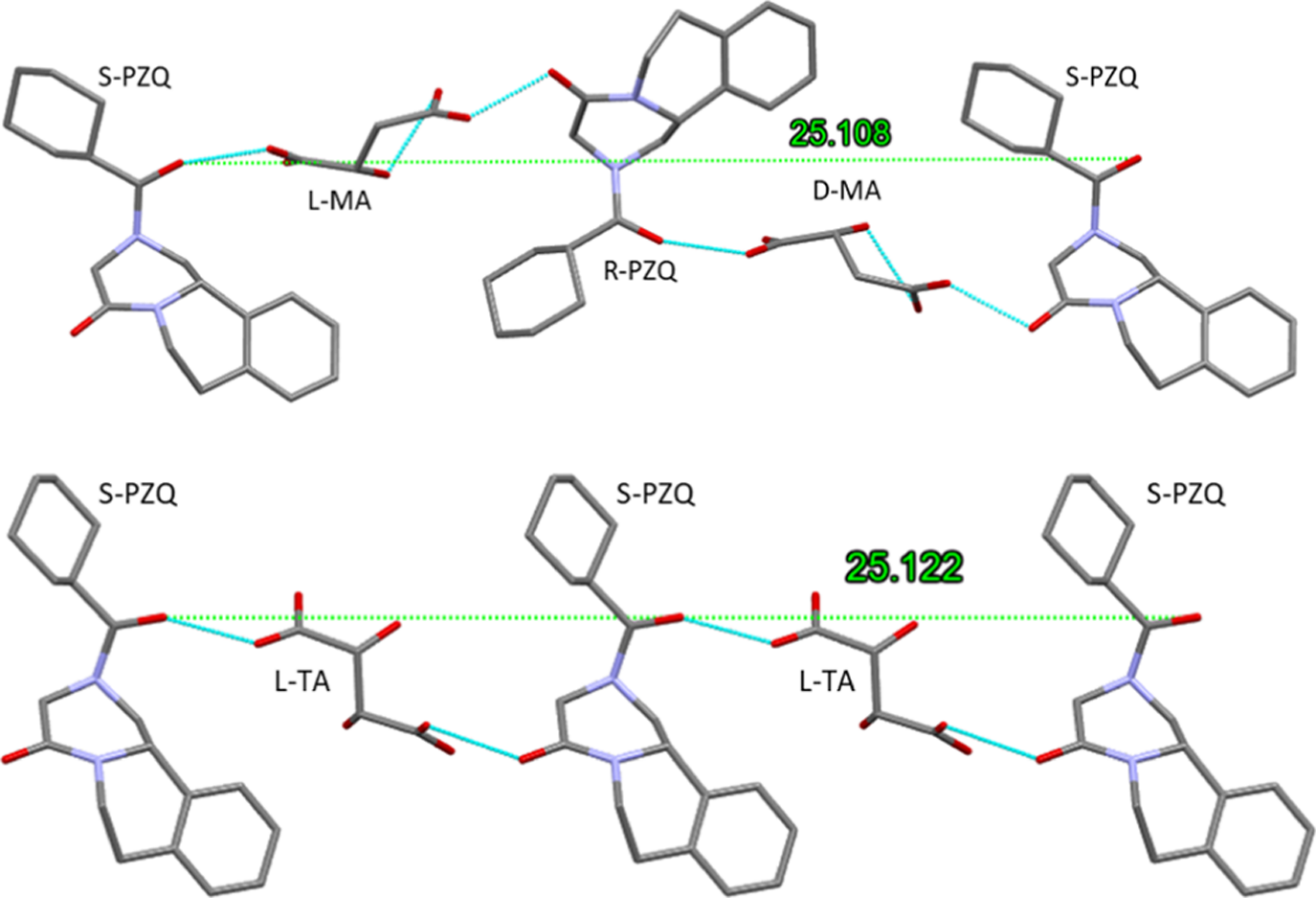
Crystal structure for R-PZQ/S-PZQ/d-MA/l-MA (**3**), top, and R-PZQ/S-PZQ/d-TA/l-TA (**4**), bottom.

PZQ is known to cocrystallize with a number of short aliphatic dicarboxylic acids.^6,7,15^ The 1:1 co-crystallization of the racemic mixture of PZQ with l-MA, either by solvent evaporation or mechanochemically, resulted in a white, microcrystalline material. Powder X-ray diffraction (PXRD) analysis reveals that the product is a mixture of two known diastereomeric cocrystals: R-PZQ/l-MA (**1**) and S-PZQ/l-MA (**2**). The crystallization of the same drug with a racemic mixture of MA afforded a four-component phase instead: R-PZQ/S-PZQ/d-MA/l-MA (**3**). Single crystals of the product could not be grown of sufficient size and quality, but the structure was solved by PXRD (Table S2). In the solid, PZQ and MA molecules alternate into racemic H-bonded chains along the *b* axis of the orthorhombic *Pbca* unit cell, with the acidic oxygen of the carboxylic acid bridging between two different carbonyl oxygen atoms of two PZQ racemes. Such a monodentate synthon has been previously described as a “type III” by Herrera-Ruiz and Höpfl.^33^ The hydroxyl group of MA acts as a H-bond donor in an intramolecular H-bond for the adjacent carboxylic group (Figures 1 and S2), while it acts as an acceptor in an additional H-bond with another MA from the nearest chains that are aligned in an antiparallel orientation in the crystal.

A similar four-component phase is obtained by repeating the co-crystallization of PZQ with a racemic mixture of TA: R-PZQ/S-PZQ/d-TA/l-TA (**4**). Once again, the structure was solved from PXRD data. In this case, chains of S-PZQ and l-TA extend along the *a* axis of the triclinic *P*1̅ cell and alternate with homologous chains of R-PZQ and d-TA that are kept together by the same monodentate interaction between the acidic group of the TA and the carbonyl group of the PZQ (Figure 1). In **4**, adjacent homochiral chains are held together by intermolecular H-bonds involving hydroxyl groups (Figure S3). Despite such substantial difference, a common alternance of the PZQ and acid is observed with an equivalent periodicity existing in the two systems (25.11 and 25.12 Å, respectively).

A structure could not be determined for the mechanochemical co-crystallization of R/S-PZQ and enantiopure d-TA. Therefore, it is not clear whether the product consists of a single three-component cocrystal (R-PZQ/S-PZQ/d-TA) or, as in the case of MA, a mixture of diastereomer cocrystals: R-PZQ/d-TA and S-PZQ/d-TA, though further analysis suggests that a single phase may be present (vide infra). For convenience, this product will be referred to as **5**.

**Figure 2:**
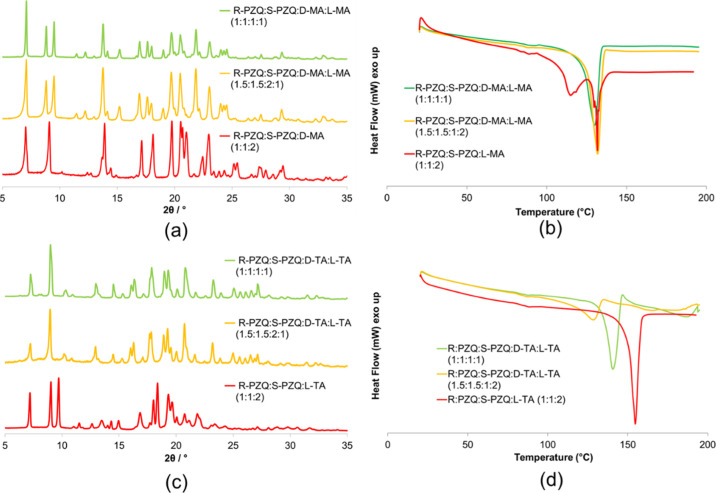
(a) PXRD patterns for the series of R-PZQ/S-PZQ/d-MA/l-MA; (b) DSC thermograms for the series of R-PZQ/S-PZQ/d-MA/l-MA; (c) PXRD patterns for the series of R-PZQ/S-PZQ/d-TA/l-TA; and (d) DSC thermograms for the series of R-PZQ/S-PZQ/d-TA/l-TA.

The knowledge of easy substitution between MA and TA prompted the attempt of a solid solution of R/S-PZQ with varied acid compositions.^23^ Ball-milling 1 equiv of R/S-PZQ with a scalemic mixture of d-MA and l-MA in a 1:2 ratio (i.e., R-PZQ/S-PZQ/d-MA/l-MA = 1.5:1.5:1:2) shows no sign of **1** and **2**. PXRD reveals a diffraction pattern that is substantially identical to that of **3** (Figure 2a), pointing toward the realization of a solid solution (**SS 3**). DSC was employed to confirm the crystallographic data: the physical mixture of **1** and **2** shows two endothermic peaks (melting) centered at 115.0 and 131.7 °C, whereas the melting point of pure PZQ was tabulated as 136 °C. As the second MA enantiomer is introduced, a single, broader endotherm event is observed between 120 and 130 °C (peak at 130.0 °C), confirming the realization of a single phase and the crystallographic evidence for a single, homogeneous phase (Figure 2b). The isolation of **SS 3** implies the mutual substitution of l-MA with its enantiomer d-MA. Similar result could be repeated when the racemic mixture of PZQ was ground with d-TA and l-TA in a 3:1:2 ratio (i.e., R-PZQ/S-PZQ/d-TA/l-TA = 1.5:1.5:1:2), although the elevated baseline (around 8°) suggested the presence of an amorphous character. Once again, the diffraction pattern coincides with that of **4** (**SS 4**) (Figure 2c). Thermal analysis shows a single peak throughout the R-PZQ/S-PZQ/d-TA/l-TA series, confirming the realization of the solid solution (Figure 2c). Notably, the endotherm onset is highest for **5**, viz, when the enantiopure acid is present.

**Figure 3:**
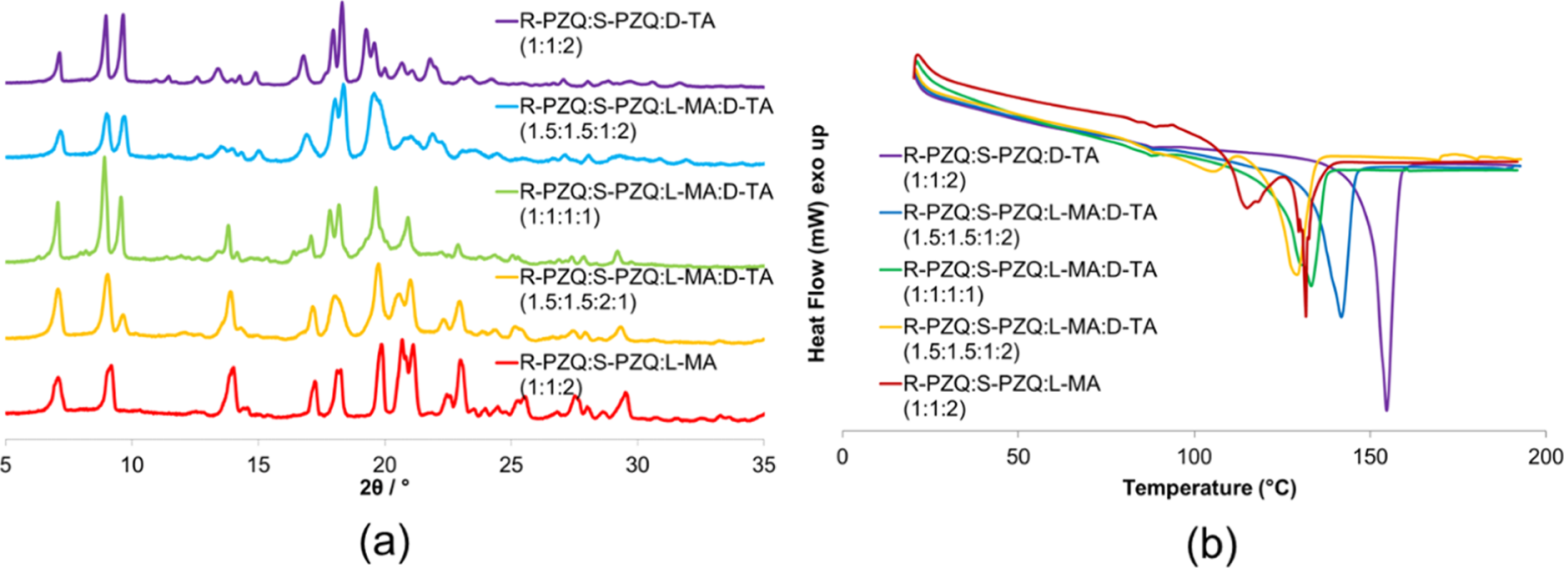
(a) PXRD patterns for the series of R-PZQ/S-PZQ/l-MA/d-TA. (b) DSC thermograms for the series of R-PZQ/S-PZQ/l-MA/d-TA.

Liquid-assisted grinding of R/S-PZQ with homochiral d-TA and l-MA, in a 1.5:1.5:1:2 ratio, did not afford a solid solution but a mixture of the PZQ/MA diastereomers (**1** and **2**) and the unknown product **5**. When the d-TA/l-MA ratio exceeds one, the diffraction peaks that are associated with **1** and **2** disappear (see in the 15–25° range), and the pattern resembles the one of **5** (Figure 3a). Once again, DSC confirms the crystallographic evidence: at 1:1:1:1 composition, the two endotherms for **1** and **2** merge in a single one (Figure 3b).

**Figure 4:**
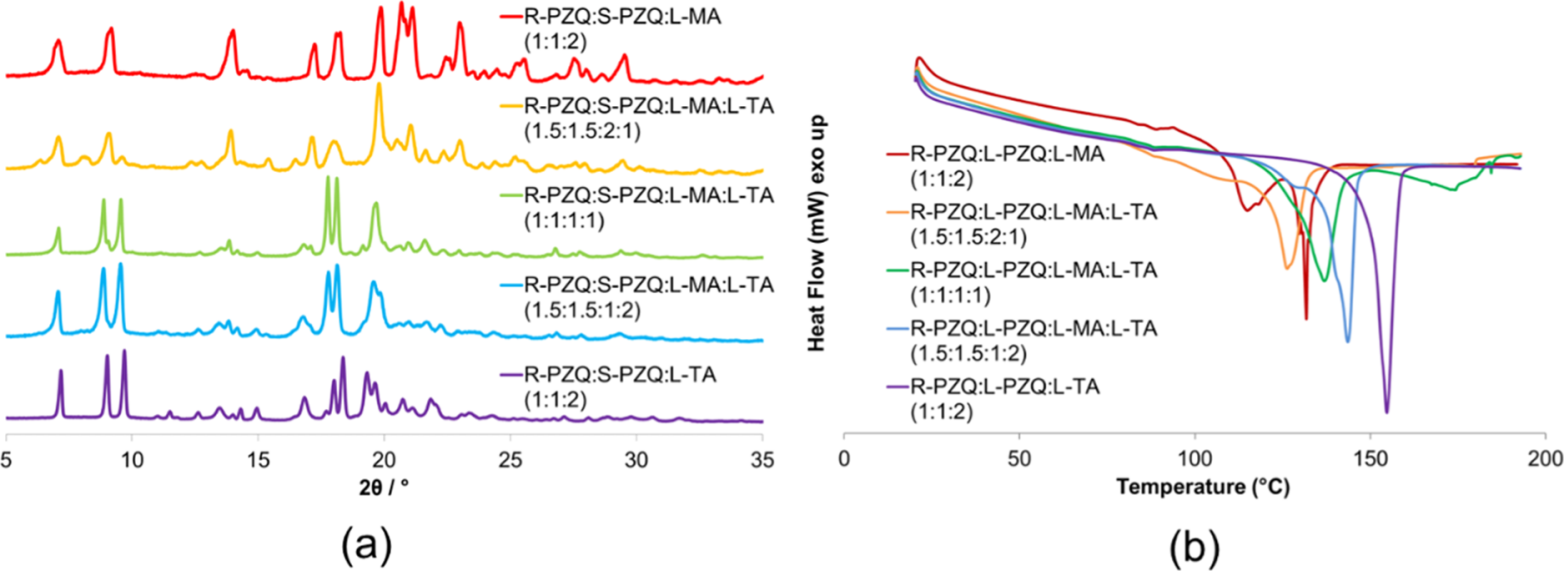
(a) PXRD patterns for the series of R-PZQ/S-PZQ/l-MA/l-TA. (b) DSC thermograms for the series of R-PZQ/S-PZQ/l-MA/l-TA.

PXRD data show another crystalline phase, whose diffraction pattern resembles that of **5**, when racemic PZQ is ball-milled with the pseudoracemate (1:1 mixture) of l-MA and l-TA (Figure 4a). The same phase persists if the amount of l-TA exceeds that of l-MA, suggesting that a four-component cocrystalline solid solution (**SS 5**) is obtained at a high TA content. The presence of diffraction peaks of **1** and **2** only appears at a higher ratio of l-MA. The product from mechanical grinding of R-PZQ, S-PZQ, l-MA, and l-TA in a 1.5:1.5:2:1 ratio shows two endotherm peaks, as for the diastereomeric mixture of **1** and **2**, confirming the PXRD evidence of a physical mixture (Figure 4b). Both thermal events are broader and occur at slightly lower temperature, which may indicate the presence of impurities in the solid forms (i.e., limited dissolution). A single endothermal melting occurs at the 1:1:1:1 composition and for a high value of TA.

Five-component solid solutions seem also possible by substituting part of l-MA with d-MA in **5** (to obtain R-PZQ/S-PZQ/d-MA/l-MA/l-TA) or part of d-TA with l-MA in **4** (to obtain R-PZQ/S-PZQ/l-MA/d-TA/l-TA), though in the latter case, extra peaks appear that could not be indexed. The solid solutions are confirmed by PXRD at least up to 1:1:0.5:0.5:1 and to 1:1:0.5:1:0.5 composition (ESI). Instead, physical mixtures were obtained while attempting the substitution of d-MA with l-TA in the cocrystal **3** (to obtain R-PZQ/S-PZQ/d-MA/l-MA/l-TA) and the solid solution with the six components together. A table and graphical summary of the solid-state landscape, as inferred from the co-crystallization screening, are provided in Supporting Information Table S1 and Figure S4.

**Figure 5:**
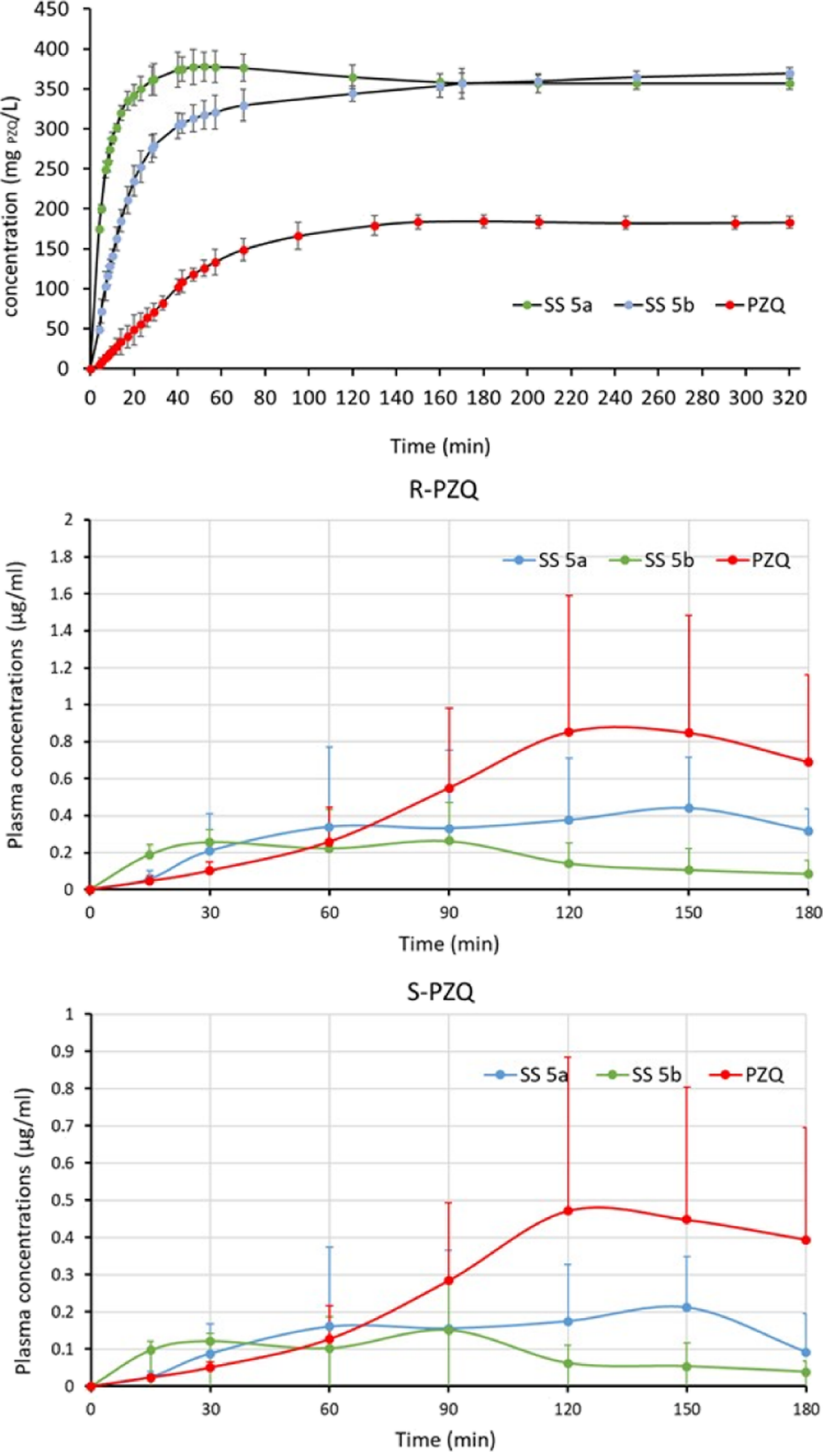
In vitro dissolution kinetics in water (top); plasma profiles of R-PZQ (middle); and plasma profiles of S-PZQ (bottom) for pure PZQ (red), **SS 5a**, R-PZQ/S-PZQ/l-MA/l-TA (1:1:1:1) (blue), and **SS 5b**, R-PZQ/S-PZQ/l-MA/d-TA (1:1:1:1) (green).

In addition to the structural characterization by PXRD and DSC, **SS 5** was further analyzed for solubility. An increase of up to 4 times depending on the relative composition was noticed (Table 1). The solubility advantage was confirmed by in vitro solubilization kinetics and non-sink conditions (Figure 5, top). The multicomponent samples show a higher dissolution rate than pure PZQ possibly due to a metastable character of these solid solutions. Indeed, in both cases, PXRD analysis reveals a solvent-mediated phase transformation, although the new phase(s) could not be identified (Figures S7 and S8). At the same time, the solubility advantage is maintained over time, suggesting that the dicarboxylic acids might stabilize the solvation of PZQ. In any case, R-PZQ/S-PZQ/l-MA/l-TA (1:1:1:1), **SS 5a**, solubilized double the amount of PZQ, whereas R-PZQ/S-PZQ/l-MA/d-TA (1:1:1:1), **SS 5b**, displayed even better performance for the first 2 h of analysis with a maximum of about 380 mg/L (Figure 5) before the onset of reprecipitation (spring-parachute effect).

**Table 1:** Equilibrium Solubility (Expressed as mg/L) at pH 7.4 (25 °C) of Different Samples in Comparison to That of Commercial PZQ

	X-TA = d-TA	X-TA = l-TA
R-PZQ/S-PZQ/**X-TA** (1:1:1)	330	340
R-PZQ/S-PZQ/l-MA/**X-TA** (1.5:1.5:2:1)	310	380
R-PZQ/S-PZQ/l-MA/**X-TA** (1:1:1:1)	370	720
R-PZQ/S-PZQ/l-MA/**X-TA** (1.5:1.5:2:1)	285	375
R-PZQ/S-PZQ/**X-MA** (1:1:2)	240	
R-PZQ/S-PZQ (1:1)	190	

For longer observation time, the performances of the two ternary samples are superimposable (ranging about 350 mg/L). Such solubility advantage is comparable to what is generally achievable for poorly soluble drugs via cocrystallization.^34^ In order to evaluate whether the increase in solubility and dissolution translates in higher oral bioavailability, in vivo pilot tests were performed on selected samples. **SS 5a** and **SS 5b** and commercial PZQ were orally administered to rats. The samples were administered as a powder in mini-capsules at a dose of 89.5 mg/kg (see experimental details). When compared to methods used in previous studies,^32^ such a procedure prevents regurgitation, simplifies the administration of the correct dose, and avoids the use of excipients or solvents that may interfere with the active pharmaceutical ingredient absorption or with animal physiology. Here, the microcapsule dosage form provides additional advantages: it masks the well-known PZQ bitter taste,^35^ thus circumventing the revulsion of the animal to the drug, and permits the comparison of active ingredient the in the marketed dosage forms. Plasma concentrations as a function of time are presented in Figure 5 for each PZQ isomer, whereas the main pharmacokinetic (PK) parameters, as calculated by means of PK SOLVER,^36^ are listed in Table 2.

**Table 2:** Main PK Parameters after Oral Administration of the Three Samples as Mini-Capsules to Rats (Mean ± S.D., *n* = 4)

*R*-isomer	PZQ	**SS 5b**	**SS 5a**
*T*_max_ (min)	97.5 ± 45.0	52.5 ± 28.7	105.0 ± 71.4
*C*_max_ (μg/mL)	0.9 ± 0.7	0.4 ± 0.2	0.6 ± 0.3
AUC_0–180min_ (μg/mL*min)	83.4 ± 62.4	33.4 ± 20.3	57.6 ± 45.6

*S*-isomer	PZQ	**SS 5b**	**SS 5a**
*T*_max_ (min)	90.0 ± 60.0	48.8 ± 33.2	127.5 ± 51.2
*C*_max_ (μg/mL)	0.5 ± 0.4	0.2 ± 0.1	0.3 ± 0.2
AUC_0–180min_ (μg/mL*min)	45.1 ± 34.2	16.2 ± 8.1	26.4 ± 20.8

We note that this pilot study involves only four animals per sample, preventing adequate statistical comparison between the average values of the PK parameters. At the same time, this preliminary survey highlights the promising characteristics of PZQ mixed crystals. In particular, the AUC_0–180min_ values suggest an increased bioavailability of the drug, although the documented variability makes it difficult to draw definitive conclusions. Plasma profiles of the samples are visibly different. In fact, the mixed crystal previously showing the fastest dissolution, sample **SS 5b**, also shows a faster in vivo absorption than pure PZQ and sample **SS 5a**. At the early time points (0–60 min), this sample provides the highest blood concentrations for both PZQ isomers, and after this period, it enables an almost constant drug concentration over a period of 150 min, whereas the blood concentration for the pure drug continues growing. Sample **SS 5b** (administered at the dose of 89.5 mg/kg) provided a *T*_max_ (Table 2) analogous to that previously reported for 50–100 mg/kg oral doses in aqueous suspensions to mice,^32^ testifying that this solid solution promotes a rapid dispersion/dissolution in physiologic media, similar to the aqueous suspension case.

On the other hand, when the drug is given as a pure racemic powder in the form of mini-capsules, the PZQ PK parameters are remarkably different from those previously reported for the pure drug in the usual administration form (aqueous suspension)^32^ in mice, underlying the importance of the dosage form and confirming the previously mentioned advantages of the mini-capsule administration. The variability of drug concentrations remains high, especially at the last sampling points, and does not permit a proper comparison of the performances of the pure drug and **SS 5a** sample, highlighting the need for further studies.

## 4 Conclusions

Racemic PZQ presents a complex solid form landscape when milled with an equivalent amount of MA and/or TA in their enantiopure, scalemic, or racemic stoichiometry. Indeed, mutual substitution of both enantiomers of MA and TA has been demonstrated in spite of different chirality and H-bond capability of the coformers. Newly characterized cocrystals confirm a common structure in which the dicarboxylic acids bridge between the drug molecules. We speculate that such arrangement, together with the relative size of the molecules, precludes the realization of additional strong H-bond interactions, and it is responsible for the observed cocrystalline solid solutions. The mixed crystals possess a higher solubility and faster dissolution rate than pure PZQ. We speculate that these properties are a consequence of both the metastable character of the solid solutions and the solvating effect of the coformers. A novel method which uses mini-capsules for the administration of solid samples to rats shows that such solubility advantage translates in an earlier and more constant absorption of the drug in the case of sample **SS 5B**, though the overall bioavailability remains similar to that of pure PZQ.

In recent years, there has been an increasing interest in the development of chemical strategies to obtain drugs with improved solubility. In fact, solid forms with higher solubility might enable better pharmacological therapies with reduce dose and side effects. This work demonstrates that cocrystalline solid solutions can further increase the solubility and absorption advantages of stoichiometric cocrystals. Moreover, given the widespread use of MA and TA as drug conformers and their ability to mix into solid solutions, the strategy demonstrated in this work might find application in other systems too.

## Supporting Information Available

Crystallographic details for **3** and **4**; difference plot for the Rietveld refinement of **3** and **4**; and PXRD patterns for the five-component solid solutions and for the sample precipitated during the solubility studies (PDF)

## References

[ref1] Meyer T.; Sekljic H.; Fuchs S.; Bothe H.; Schollmeyer D.; Miculka C. Taste, A New Incentive to Switch to (R)-Praziquantel in Schistosomiasis Treatment. PLoS Neglected Trop. Dis. 2009, 3, e357 10.1371/journal.pntd.0000357. PMC2614124 19159015

[ref2] FDA. ANDAs: Size, Shape, and Other Physical Attributes of Generic Tablets and Capsules, Guidance for Industry; U.S. Department of Health and Human Services, Ed.; Silver Spring: MD, USA, 2015.

[ref3] Li N.; Taylor L. S. Tailoring supersaturation from amorphous solid dispersions. J. Controlled Release 2018, 279, 114–125. 10.1016/j.jconrel.2018.04.014. PMC5972073 29654798

[ref4] Babu N. J.; Nangia A. Solubility Advantage of Amorphous Drugs and Pharmaceutical Cocrystals. Cryst. Growth Des. 2011, 11, 2662–2679. 10.1021/cg200492w.

[ref5] Bond A. D. What is a co-crystal?. CrystEngComm 2007, 9, 833–834. 10.1039/b708112j.

[ref6] Sánchez-Guadarrama O.; Mendoza-Navarro F.; Cedillo-Cruz A.; Jung-Cook H.; Arenas-García J. I.; Delgado-Díaz A.; Herrera-Ruiz D.; Morales-Rojas H.; Höpfl H. Chiral Resolution of RS-Praziquantel via Diastereomeric Co-Crystal Pair Formation with l-Malic Acid. Cryst. Growth Des. 2016, 16, 307–314. 10.1021/acs.cgd.5b01254.

[ref7] Espinosa-Lara J. C.; Guzman-Villanueva D.; Arenas-García J. I.; Herrera-Ruiz D.; Rivera-Islas J.; Román-Bravo P.; Morales-Rojas H.; Höpfl H. Cocrystals of Active Pharmaceutical Ingredients-Praziquantel in Combination with Oxalic, Malonic, Succinic, Maleic, Fumaric, Glutaric, Adipic, And Pimelic Acids. Cryst. Growth Des. 2013, 13, 169–185. 10.1021/cg301314w.

[ref8] Salas-Zúñiga R.; Mondragón-Vásquez K.; Alcalá-Alcalá S.; Lima E.; Höpfl H.; Herrera-Ruiz D.; Morales-Rojas H. Nanoconfinement of a Pharmaceutical Cocrystal with Praziquantel in Mesoporous Silica: The Influence of the Solid Form on Dissolution Enhancement. Mol. Pharm. 2022, 19, 414–431. 10.1021/acs.molpharmaceut.1c00606. 34967632

[ref9] Borrego-Sánchez A.; Viseras C.; Aguzzi C.; Sainz-Díaz C. I. Molecular and crystal structure of praziquantel. Spectroscopic properties and crystal polymorphism. Eur. J. Pharm. Sci. 2016, 92, 266–275. 10.1016/j.ejps.2016.04.023. 27108679

[ref10] Zanolla D.; Perissutti B.; Passerini N.; Chierotti M. R.; Hasa D.; Voinovich D.; Gigli L.; Demitri N.; Geremia S.; Keiser J.; Cerreia Vioglio P.; Albertini B. A new soluble and bioactive polymorph of praziquantel. Eur. J. Pharm. Biopharm. 2018, 127, 19–28. 10.1016/j.ejpb.2018.01.018. 29409939

[ref11] Zanolla D.; Perissutti B.; Vioglio P. C.; Chierotti M. R.; Gigli L.; Demitri N.; Passerini N.; Albertini B.; Franceschinis E.; Keiser J.; Voinovich D. Exploring mechanochemical parameters using a DoE approach: Crystal structure solution from synchrotron XRPD and characterization of a new praziquantel polymorph. Eur. J. Pharm. Sci. 2019, 140, 105084 10.1016/j.ejps.2019.105084. 31626966

[ref12] Saikia B.; Seidel-Morgenstern A.; Lorenz H. Role of Mechanochemistry in Solid Form Selection and Identification of the Drug Praziquantel. Cryst. Growth Des. 2021, 21, 5854–5861. 10.1021/acs.cgd.1c00736.

[ref13] Zanolla D.; Hasa D.; Arhangelskis M.; Schneider-Rauber G.; Chierotti M. R.; Keiser J.; Voinovich D.; Jones W.; Perissutti B. Mechanochemical Formation of Racemic Praziquantel Hemihydrate with Improved Biopharmaceutical Properties. Pharmaceutics 2020, 12, 289 10.3390/pharmaceutics12030289. 32210129 PMC7151222

[ref14] Zanolla D.; Gigli L.; Hasa D.; Chierotti M. R.; Arhangelskis M.; Demitri N.; Jones W.; Voinovich D.; Perissutti B. Mechanochemical Synthesis and Physicochemical Characterization of Previously Unreported Praziquantel Solvates with 2-Pyrrolidone and Acetic Acid. Pharmaceutics 2021, 13, 1606 10.3390/pharmaceutics13101606. 34683899 PMC8540171

[ref15] Devogelaer J.-J.; Charpentier M. D.; Tijink A.; Dupray V.; Coquerel G.; Johnston K.; Meekes H.; Tinnemans P.; Vlieg E.; ter Horst J. H.; de Gelder R. Cocrystals of Praziquantel: Discovery by Network-Based Link Prediction. Cryst. Growth Des. 2021, 21, 3428–3437. 10.1021/acs.cgd.1c00211. PMC8276530 34276256

[ref16] Roy L.; Lipert M. P.; Rodriguez-Hornedo N. Chapter 11 Co-crystal Solubility and Thermodynamic Stability. In Pharmaceutical Salts and Co-crystals; The Royal Society of Chemistry, 2012; pp 247–279.

[ref17] Lusi M. Engineering Crystal Properties through Solid Solutions. Cryst. Growth Des. 2018, 18, 3704–3712. 10.1021/acs.cgd.7b01643.

[ref18] Lusi M. A rough guide to molecular solid solutions: design, synthesis and characterization of mixed crystals. CrystEngComm 2018, 20, 7042–7052. 10.1039/c8ce00691a.

[ref19] Lestari M.; Lusi M. A mixed molecular salt of lithium and sodium breaks the Hume-Rothery rules for solid solutions. Chem. Commun. 2019, 55, 2297–2300. 10.1039/c8cc09850f. 30714594

[ref20] Schur E.; Nauha E.; Lusi M.; Bernstein J. Kitaigorodsky Revisited: Polymorphism and Mixed Crystals of Acridine/Phenazine. Chem.—Eur. J. 2015, 21, 1735–1742. 10.1002/chem.201404321. 25417965

[ref21] Lusi M.; Vitorica-Yrezabal I. J.; Zaworotko M. J. Expanding the Scope of Molecular Mixed Crystals Enabled by Three Component Solid Solutions. Cryst. Growth Des. 2015, 15, 4098–4103. 10.1021/acs.cgd.5b00685.

[ref22] Verma V.; Bordignon S.; Chierotti M. R.; Lestari M.; Lyons K.; Padrela L.; Ryan K. M.; Lusi M. Cortisone and Cortisol Break H-bonding Rules toMake a Drug-Prodrug Solid Solution. IUCrJ 2020, 7, 1124 10.1107/S2052252520013263. PMC7642785 33209323

[ref23] Cruz-Cabeza A. J.; Lestari M.; Lusi M. Cocrystals Help Break the “Rules” of Isostructurality: Solid Solutions and Polymorphism in the Malic/Tartaric Acid System. Cryst. Growth Des. 2018, 18, 855–863. 10.1021/acs.cgd.7b01321.

[ref24] Peterson M.; Hickey M. B.; Oliveira M.; Almarsson Ö.; Remenar J. Mixed co-crystals and pharmaceutical compositions comprising the same. U.S. Patent 7,671,093 B2, 2010.

[ref25] Altomare A.; Cuocci C.; Giacovazzo C.; Moliterni A.; Rizzi R.; Corriero N.; Falcicchio A. EXPO2013: a kit of tools for phasing crystal structures from powder data. J. Appl. Crystallogr. 2013, 46, 1231–1235. 10.1107/s0021889813013113.

[ref26] Stewart J. J. P. Optimization of parameters for semiempirical methods VI: more modifications to the NDDO approximations and re-optimization of parameters. J. Mol. Model. 2013, 19, 1–32. 10.1007/s00894-012-1667-x. 23187683 PMC3536963

[ref27] Eason T.; Ramirez G.; Clulow A. J.; Salim M.; Boyd B. J. Revisiting the Dissolution of Praziquantel in Biorelevant Media and the Impact of Digestion of Milk on Drug Dissolution. Pharmaceutics 2022, 14, 2228 10.3390/pharmaceutics14102228. 36297662 PMC9609124

[ref28] Hasa D.; Perissutti B.; Cepek C.; Bhardwaj S.; Carlino E.; Grassi M.; Invernizzi S.; Voinovich D. Drug salt formation via mechanochemistry: the case study of vincamine. Mol. Pharm. 2013, 10, 211–224. 10.1021/mp300371f. 23186380

[ref29] Lax E. R.; Militzer K.; Trauschel A. A simple method for oral administration of drugs in solid form to fully conscious rats. Lab. Anim. 1983, 17, 50–54. 10.1258/002367783781070894. 6865310

[ref30] Battaglia L.; Serpe L.; Muntoni E.; Zara G.; Trotta M.; Gallarate M. Methotrexate-loaded SLNs prepared by coacervation technique: in vitro cytotoxicity and in vivo pharmacokinetics and biodistribution. Nanomedicine 2011, 6, 1561–1573. 10.2217/nnm.11.52. 22011315

[ref31] Meister I.; Leonidova A.; Kovač J.; Duthaler U.; Keiser J.; Huwyler J. Development and validation of an enantioselective LC-MS/MS method for the analysis of the anthelmintic drug praziquantel and its main metabolite in human plasma, blood and dried blood spots. J. Pharm. Biomed. Anal. 2016, 118, 81–88. 10.1016/j.jpba.2015.10.011. 26517852

[ref32] Lombardo F. C.; Perissutti B.; Keiser J. Activity and pharmacokinetics of a praziquantel crystalline polymorph in the Schistosoma mansoni mouse model. Eur. J. Pharm. Biopharm. 2019, 142, 240–246. 10.1016/j.ejpb.2019.06.029. 31265895

[ref33] Rodríguez-Ruiz C.; Salas-Zúñiga R.; Sánchez-Guadarrama M. O.; Delgado-Díaz A.; Herrera-Ruiz D.; Morales-Rojas H.; Höpfl H. Structural, Physicochemical, and Biopharmaceutical Properties of Cocrystals with RS- and R-Praziquantel–Generation and Prolongation of the Supersaturation State in the Presence of Cellulosic Polymers. Cryst. Growth Des. 2022, 22, 6023–6038. 10.1021/acs.cgd.2c00661.

[ref34] Childs S. L.; Chyall L. J.; Dunlap J. T.; Smolenskaya V. N.; Stahly B. C.; Stahly G. P. Crystal Engineering Approach To Forming Cocrystals of Amine Hydrochlorides with Organic Acids. Molecular Complexes of Fluoxetine Hydrochloride with Benzoic, Succinic, and Fumaric Acids. J. Am. Chem. Soc. 2004, 126, 13335–13342. 10.1021/ja048114o. 15479089

[ref35] Zanolla D.; Bertoni S.; Passerini N.; Albertini B.; Zingone G.; Perissutti B. From Bitter to Sweet: a preliminary study towards a patient-friendly Praziquantel dosage form. C. R. Chim. 2022, 25, 179–188. 10.5802/crchim.188.

[ref36] Zhang Y.; Huo M.; Zhou J.; Xie S. PKSolver: An add-in program for pharmacokinetic and pharmacodynamic data analysis in Microsoft Excel. Comput. Methods Programs Biomed. 2010, 99, 306–314. 10.1016/j.cmpb.2010.01.007. 20176408

