# Supplementary material for: Co-Crystalline Solid Solution Affords a High-Soluble and Fast-Absorbing Form of Praziquantel
Source: Mol Pharm. 2023 Mar 8;20(4):2009–16. doi: 10.1021/acs.molpharmaceut.2c00984 (PMC10074383; doi:10.1021/acs.molpharmaceut.2c00984)
Supplement: Supplementary file 1 — mp2c00984_si_001.pdf [file mp2c00984_si_001.pdf]

SUPPLEMENTARY INFORMATION

A co-crystalline solid solution affords a high-soluble and fast-absorbing form of praziquantel

Chiara Cappuccino[1], Enrico Spoletti[1], Fiammetta Renni[1,2], Elisabetta Muntoni[3], Jennifer Keiser[4-5], Dario Voinovich[2], Beatrice Perissutti[2], Matteo Lusi[1]*

Reaction summary Page 2

Crystallographic information Page 3

Rietveld refinement Page 4

5-component ball milling Page 5

Assessment of solution-mediated transformations Page 6

Reaction Summary

Table S1: Summary of the product identified in this work

R-PZQ	S-PZQ	D-MA	L-MA	D-TA	L-TA	Product
0.5	0.5	0	1	0	0	**CC1; CC2**
1.5	1.5	1	2	0	0	**SS3**
1	1	1	1	0	0	**CC3**
0.5	0.5	0	0	0	1	**CC5**
1.5	1.5	0	0	1	2	**SS4**
1	1	0	0	1	1	**CC4**
1.5	1.5	0	2	1	0	**CC1; CC2; 5**
1	1	0	1	1	0	**SS5**
1.5	1.5	0	1	2	0	**SS5**
1.5	1.5	0	2	0	1	**CC1; CC2; 5**
1	1	0	1	0	1	**SS5**
1.5	1.5	0	1	0	2	**SS5**
2	2	1	1	0	2	**SS5**
2	2	0	1	1	2	**SS4**
2	2	1	1	1	1	**CC3 CC4**

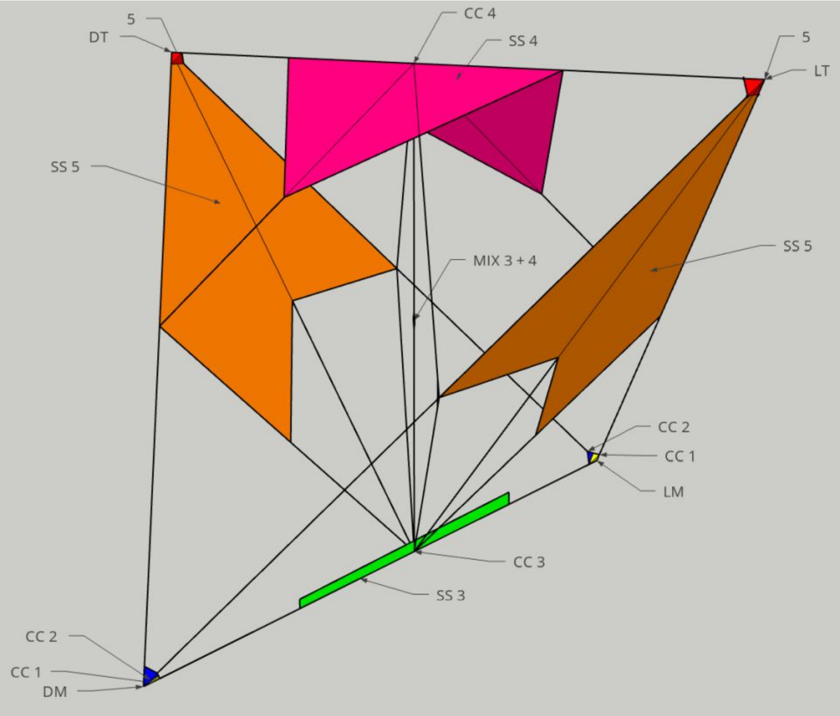

Figure S1. 3-D phase diagram for PZQ in combination with four dicarboxylic acids.

Crystallographic information

Table S1: Crystal data and structure refinement details.

	**R-PZQ:S-PZQ:D-MA:L-MA (3)**	**R-PZQ:S-PZQ:D-TA:L-TA (4)**
Empirical formula	$C_{23} H_{27} N_2 O_7$	$C_{23} H_{28} N_2 O_8$
Formula weight (g mol^{-1})	443.473	460.480
T (K)	293	293
Wavelength (Å)	1.540598	1.540598
Crystal system	orthorhombic	triclinic
Space group	P b c a	P-1
a (Å)	20.150(4)	12.561(3)
b (Å)	25.108(5)	10.749(2)
c (Å)	8.8963(16)	9.2445(17)
α (°)	90	110.316(4)
β (°)	90	95.675(4)
γ (°)	90	100.004(6)
V (Å^3)	4501.0(15)	1135.5(4)
Z	8	2
ϑ range for data collection (◦)	5° to 60°	5° to 60°
R_{int}	0.07524	0.06217
Refinement method	Rietveld	Rietveld
Number parameters	34	181
Goodness-of-fit	2.70384	2.40215
R_{wp}	0.10378	0.08239
R_{exp}	0.03838	0.03430

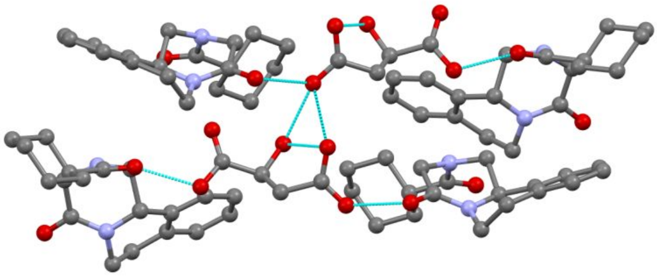

Figure S2: H-bond motif across multiple chains in R-PZQ:S-PZQ:D-MA:L-MA (**3**).

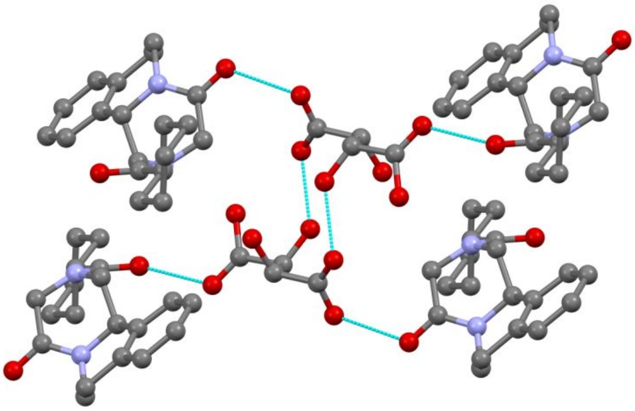

Figure S3: H-bond motif across multiple chains in R-PZQ:S-PZQ:D-TA:L-TA (**4**).

Rietveld refinement

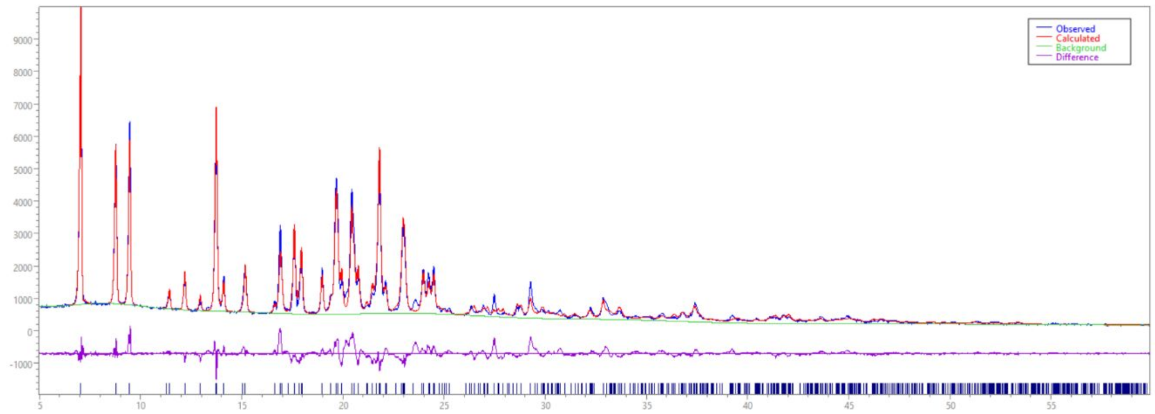

Figure S4: Rietveld refinement (red line) of R-PZQ:S-PZQ:D-MA:L-MA (**3**) compared with the experimental diffraction pattern (blue line). The purple line indicates the difference plot.

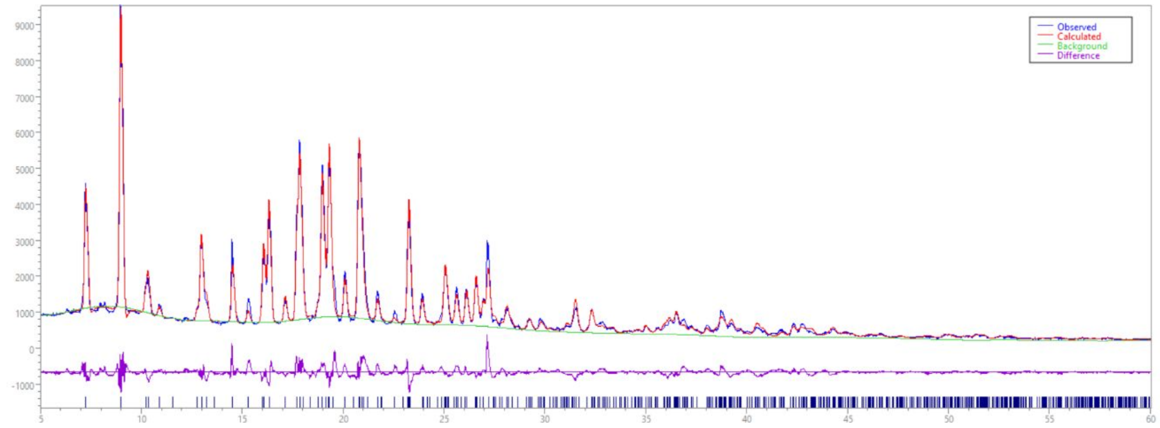

Figure S5: Rietveld refinement (red line) of R-PZQ:S-PZQ:D-TA:L-TA (**4**) compared with the experimental diffraction pattern (blue line). The purple line indicates the difference plot.

5-component ball milling

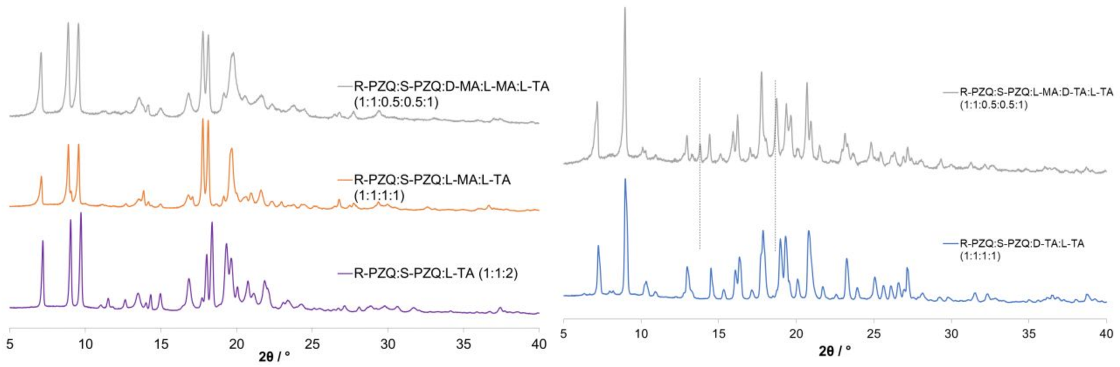

Figure S6: PXRD patterns for the 5-component solid solutions

Assessment of solution-mediated transformations

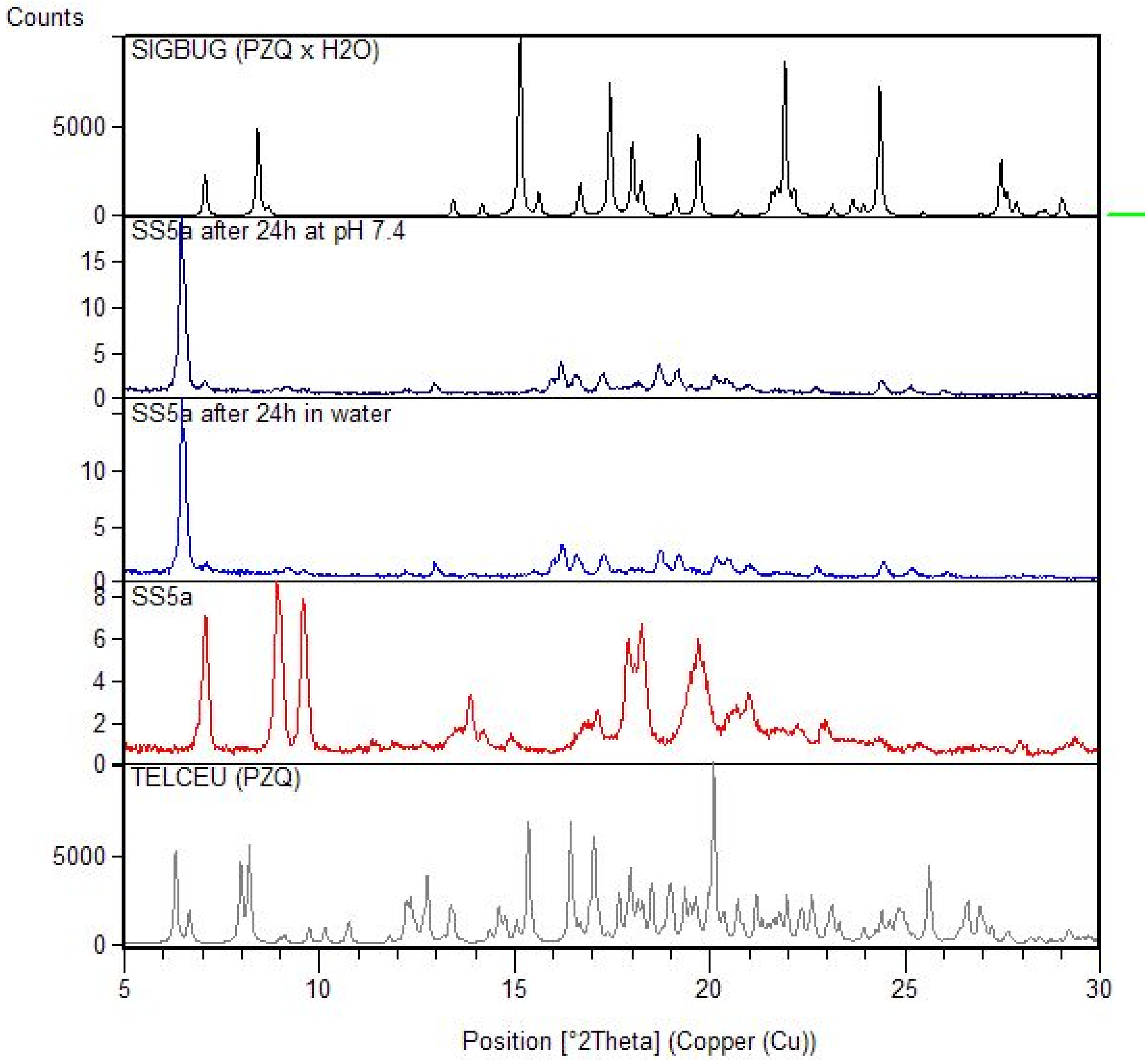

Figure S7 Comparison of PXRD patterns calculates for pure PZQ and PZQxH_2O against those measured for SS5a as synthesised, after 24h in H_2O and after 24h in a buffer solution at pH 7.4

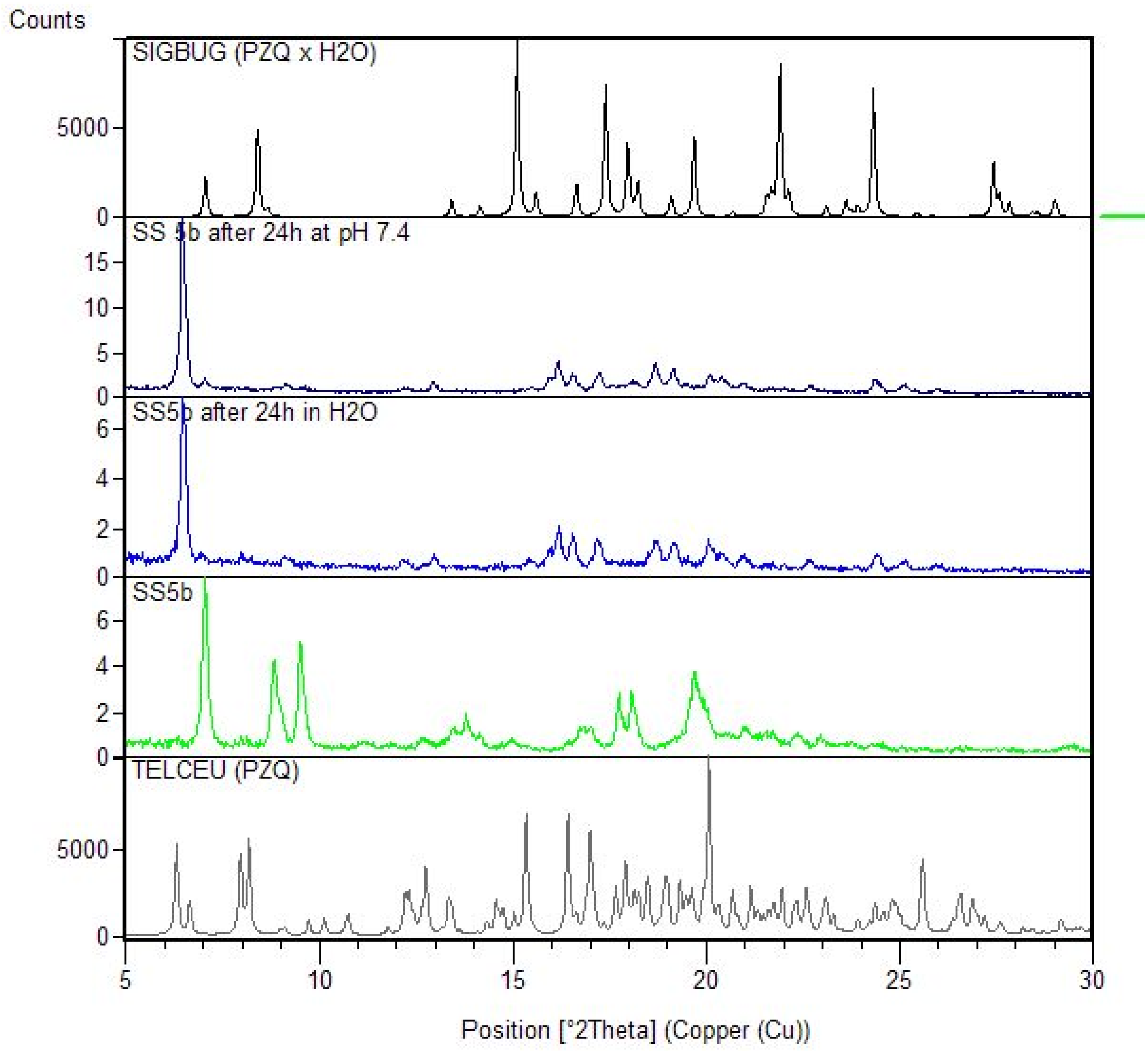

Figure S8 Comparison of PXRD patterns calculates for pure PZQ and PZQxH_2O against those measured for SS5a as synthesised, after 24h in H_2O and after 24h in a buffer solution at pH 7.4
